# Novel Akt activator SC-79 is a potential treatment for alcohol-induced osteonecrosis of the femoral head

**DOI:** 10.18632/oncotarget.16075

**Published:** 2017-03-10

**Authors:** Yi-Xuan Chen, Shi-Cong Tao, Zheng-Liang Xu, Wen-Jing Yin, Yue-Lei Zhang, Jun-Hui Yin, You-Shui Gao, Chang-Qing Zhang

**Affiliations:** ^1^ Department of Orthopedic Surgery, Shanghai Jiao Tong University Affiliated Sixth People's Hospital, Shanghai 200233, China; ^2^ Institute of Microsurgery on Extremities, Shanghai 200233, China

**Keywords:** ethanol, Akt pathway, osteonecrosis of the femoral head, BMSC, SC-79

## Abstract

Alcohol is a leading risk factor for osteonecrosis of the femoral head (ONFH). We explored the molecular mechanisms underlying alcohol-induced ONFH and investigated the protective effect of the novel Akt activator SC-79 against this disease. We found that ethanol inhibited expression of the osteogenic genes *RUNX2* and *OCN*, downregulated osteogenic differentiation, impaired the recruitment of Akt to the plasma membrane, and suppressed Akt phosphorylation at Ser473, thereby inhibiting the Akt/GSK3β/β-catenin signaling pathway in bone mesenchymal stem cells. To assess SC-79′s ability to counteract the inhibitory effect of ethanol on Akt-Ser73 phosphorylation, we performed micro-computerized tomography and immunofluorescent staining of osteopontin, osteocalcin and collagen type 1 in a rat model of alcohol-induced ONFH. We found that SC-79 injections inhibited alcohol-induced osteonecrosis. These results show that alcohol-induced ONFH is associated with suppression of p-Akt-Ser473 in the Akt/GSK3β/β-catenin signaling pathway in bone mesenchymal stem cells. We propose that SC-79 treatment to rescue Akt activation could be tested in the clinic as a potential therapeutic approach to preventing the development of alcohol-induced ONFH.

## INTRODUCTION

Osteonecrosis of the femoral head (ONFH) is a devastating bone disease in which patients experience progressive collapse of the femoral head. ONFH can be classified into traumatic and non-traumatic. Alcohol consumption is one of the leading causes of non-traumatic ONFH. Other etiologies include systemic lupus erythematous, steroid medication, disorders of lipid metabolism, and sickle-cell anemia [1–4]. The physiological mechanisms underlying alcohol-induced ONFH remain unclear [3, 5, 6].

In previous studies, decreased bone synthesis and increased bone breakdown were detected in alcoholic patients. Furthermore, chronic exposure to ethanol decreased bone mineral density (BMD) in humans [7–10]. Moreover, ethanol was able to reduce the proliferation and osteogenic differentiation of bone mesenchymal stem cells (BMSCs) [11, 12], which play help to repair and regenerate bone tissues [13]. Thus, impaired BMSC osteogenesis might contribute to alcohol-induced ONFH.

Akt is a critical kinase in the PI3-kinase (PI3K) pathway that controls bone development [14]. The structure of Akt consists of three domains, namely, the N-terminal pleckstrin homology (PH) domain, the C-terminal regulatory domain, and a central kinase domain (KD) [15, 16]. Akt activation requires the membrane interaction and phosphorylation of serine 473 (Akt-Ser473) and threonine 308 (Akt-Thr308). Upon growth factor stimulation, cytosolic Akt is recruited to the plasma membrane by the interaction between the PH domain and phosphatidylinositol 3, 4, 5-trisphosphate (PIP3) generated by PI3K [17–19]. Next, Akt-PIP3 interactions induce Akt to an open conformer through inter-domain conformational changes, which expose Thr308 and Ser473 for subsequent phosphorylation by phosphoinositide-dependent protein kinases (PDKs) [20, 21]. Phosphorylation at Thr308 and Ser473 fully activates Akt to phosphorylate various downstream kinases that modulate cell proliferation and survival, among other functions [22]. Glycogen synthesis kinase-3β (GSK3β) acts downstream of Akt and has been reported to participate in the canonical Wnt/β-catenin signaling pathway, thereby enhancing cellular osteogenesis [14, 23, 24].

Here we report our discovery that the membrane-induced interdomain conformational change of Akt could be disrupted by ethanol, resulting in dephosphorylation of Akt-Ser473, activation of GSK3β kinase, increase in β-catenin degradation, and osteogenic differentiation impairment of BMSCs. We therefore hypothesized that the ethanol-induced dephosphorylation of Akt-Ser473 contributed to the suppression of Akt/GSK3β/β-catenin signaling, leading to alcohol-induced ONFH. We employed a plethora of *in vitro* assays to test our hypothesis. Akt-membrane interaction and Ser473 phosphorylation were blocked by ethanol treatment upon IGF-1 simulation in BMSCs. Furthermore, co-administration of an Akt activator, SC-79, blocked the suppressive effects of ethanol on osteogenesis, both *in vitro* and *in vivo*. The treatment of GSK3β inhibitor also rescued the anti-osteogenesis effects of ethanol *in vitro*. Hence, we demonstrated that the downregulation of the Akt/GSK3β/β-catenin signaling pathway correlated with the suppression of osteogenic differentiation by ethanol, leading to alcohol-induced ONFH.

## RESULTS

### Ethanol impairs osteogenesis potential of BMSCs

**Figure 1:**
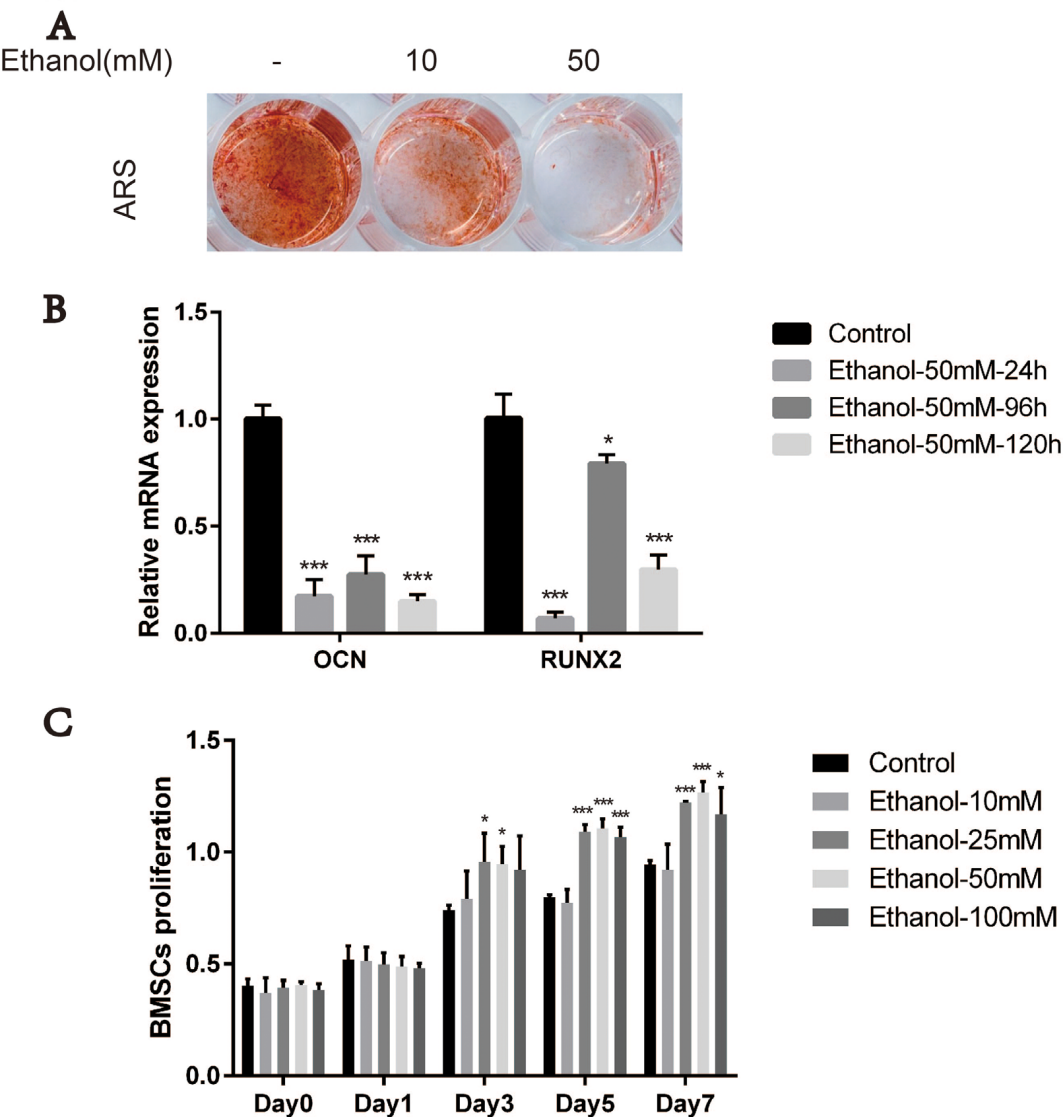
Ethanol impairs osteogenesis potential of BMSCs (**A**) Alizarin red staining (ARS) of BMSCs was reduced after 14-day incubation with osteogenic medium supplemented with ethanol. A higher concentration (50 mM) of ethanol resulted in stronger anti-osteogenic effects on BMSCs. (**B**) The mRNA expression of RUNX2 and OCN in BMSCs after 24 h, 96 h and 120 h incubation with 50 mM of ethanol was decreased compared with controls. Values are shown as mean ± SD (*N* = 3) (***, **, * indicate statistically significant differences compared to controls,***p < 0.001, **p < 0.01, *p < 0.05). (**C**) Proliferation of BMSCs incubated for 1, 3, 5 and 7 days in medium supplemented with 10 mM, 25 mM, 50 mM and 100 mM of ethanol. Ethanol treatment at different concentrations as indicated did not impair the survival and proliferation of BMSCs. Values are shown as mean ± SD (*n* = 3) (***, **, * indicate statistically significant differences compared to controls,***p < 0.001, **p < 0.01, *p < 0.05).

BMSCs are the main precursor cells for bone formation and regeneration; therefore, we first evaluated the effects of ethanol on osteogenic responses of BMSCs *in vitro*. We performed alizarin red staining (ARS) after 14 days of osteogenic induction in ethanol-containing medium. As shown in Figure 1A, the extent of calcium mineral deposition production in BMSCs treated with ethanol (10 mM) was drastically reduced compared with the control group. The anti-osteogenic effects of ethanol were stronger upon increasing its concentration (50 mM). In subsequent experiments, we used ethanol at 50 mM concentration, as suggested in previous studies [11, 25]. To explore the effect of alcohol on the expression of osteogenic genes including osteocalcin (OCN) and runt-related transcription factor 2 (RUNX2) in BMSCs, we performed qRT-PCR analysis. Our experiments showed that the expression of these genes was inhibited in BMSCs in the presence of ethanol compared with controls (Figure 1B, ***p < 0.001, **p < 0.01, *p < 0.05). Additionally, different concentrations of ethanol (10mM, 25 mM, 50 mM and 100 mM) did not impair the BMSCs survival and proliferation. On the contrary, higher ethanol concentrations(25 mM, 50 mM and 100 mM) enhanced BMSCs proliferation compared with controls (Figure 1C,***p < 0.001, **p < 0.01, *p < 0.05). These results suggested that alcohol treatment impaired BMSC osteogenesis.

### Downregulation of Akt/GSK3β/β-catenin signaling suppresses osteogenesis of ethanol-treated BMSCs

**Figure 2:**
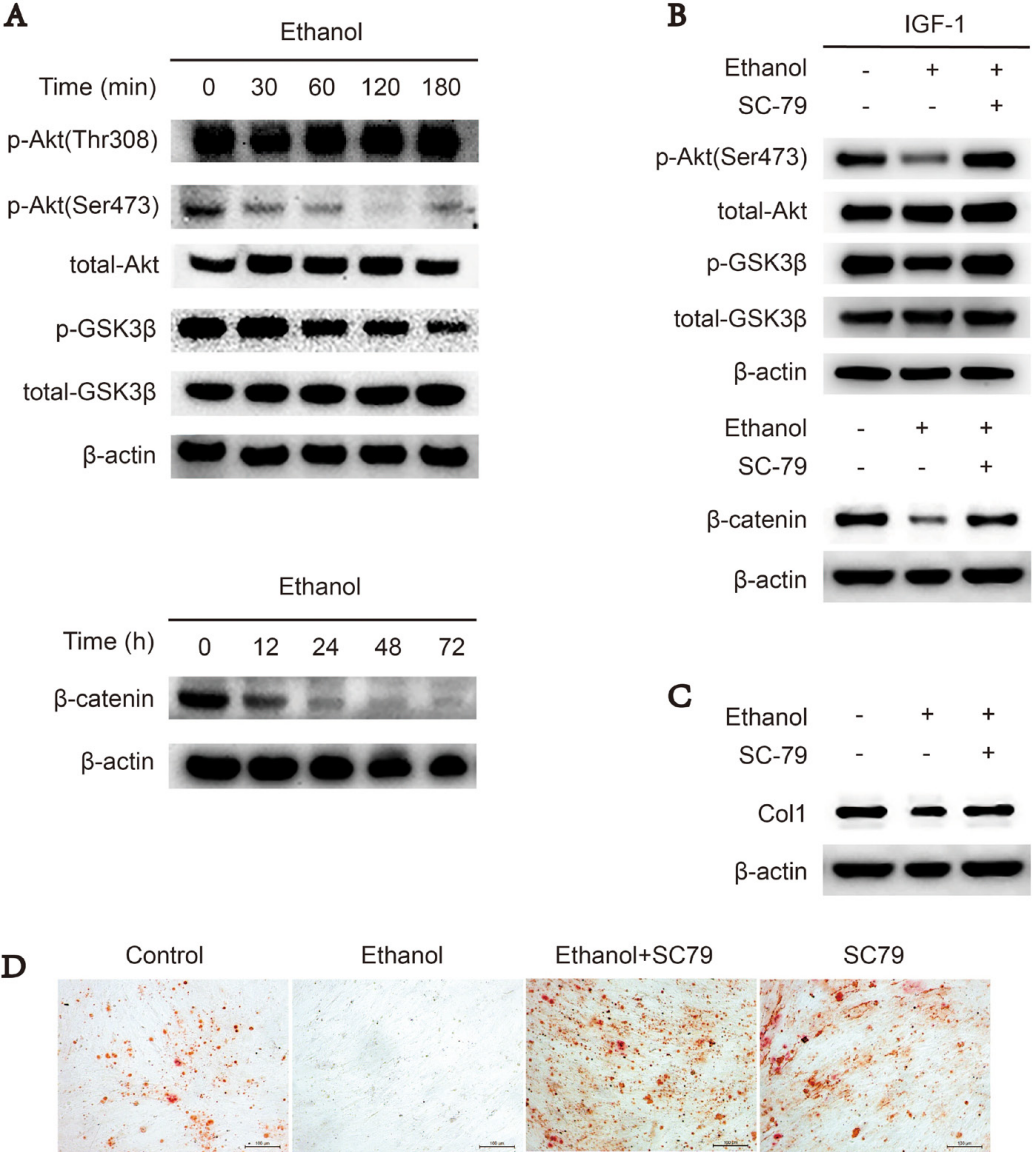
Downregulation of Akt/GSK3β/β-catenin signaling suppresses osteogenesis of ethanol-treated BMSCs (**A**) Ethanol treatment blocked p-Akt-Ser473 and the Akt/GSK3β/β-catenin signaling pathway in a time-dependent manner in BMSCs. Proteins were immunoblotted with primary antibodies against p-Akt-Ser473, p-Akt-Thr308, total-Akt, p-GSK3β, total-GSK3β and β-catenin. β-actin served as a normalization control. (**B**) SC-79 rescued the ethanol-induced inhibition of the Akt/GSK3β/β-catenin axis in BMSCs. The western blot shows p-Akt-Ser473, total-Akt, p-GSK3β and total-GSK3β, and β-catenin. β-actin served as an internal reference. (**C**) SC-79 rescued the anti-osteogenic effect of ethanol. The western blot shows COL1 and β-actin served as an internal reference. (**D**) Alizarin red staining showed that SC-79 treatment significantly increased the calcium nodules produced by BMSCs.

Akt/GSK3β/β-catenin signaling promotes cellular osteogenesis [23, 24, 26]. First, we used western blotting to assess whether ethanol could downregulate Akt/GSK3β/β-catenin signaling in BMSCs. Our results revealed that ethanol-treated BMSCs exhibited decreased levels of phosphorylated Akt-Ser473, GSK3β and the downstream target β-catenin compared to the control group, indicating Akt/GSK3β/β-catenin signaling was impaired by ethanol administration in a time-dependent manner (Figure 2A). However, p-Akt-Thr308 was not decreased by ethanol treatment (Figure 2A). When co-administrated with SC-79, an Akt activator, ethanol-dependent inhibition of Akt/GSK3β/β-catenin signaling was abolished (Figure 2B). In addition, the protein level of collagen 1 (COL1) (Figure 2C) and calcium deposit production (Figure 2D) in BMSCs were restored by co-administrated with SC-79.

### SC-79 induced cytosolic activation of Akt antagonizes the ethanol-induced inhibitory effect on Ser473 in BMSCs

**Figure 3:**
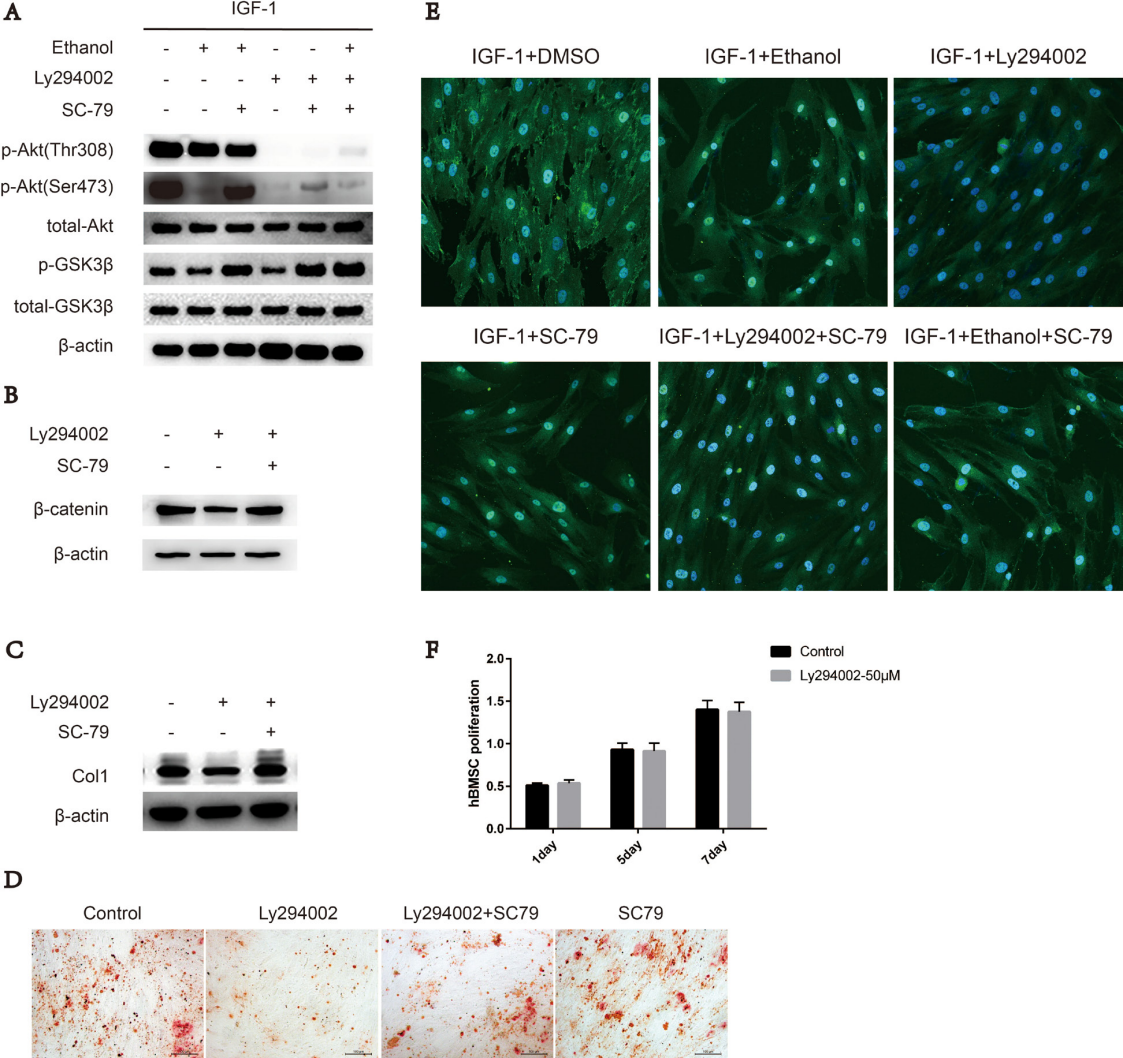
SC-79 induced cytosolic activation of Akt antagonizes the inhibitory effect of ethanol on Ser473 in BMSCs (**A**) p-Akt-Ser473 levels in BMSCs decreased upon ethanol treatment while p-Akt-Thr308 status remained the same. SC-79 rescued p-Akt-Ser473 and p-GSK3β levels, impaired by ethanol or Ly294002 treatment. The western blot shows p-Akt, total-Akt, p-GSK3β, total-GSK3β, and β-actin as internal reference. (**B**) Western blot indicated that SC-79 could rescue β-catenin, impaired by Ly294002 treatment. β-actin served as internal reference. (**C**) SC-79 rescued the anti-osteogenic effect induced by Ly294002. The western blot shows COL1 and β-actin served as an internal reference. (**D**) Alizarin red staining was performed after BMSCs treated with control, Ly294002 (50 μM), SC-79 (10 μM) or their combination for 14 days. (**E**) Ethanol, Ly294002, SC-79 and their combinations blocked the translocation of Akt from cytoplasm to plasma membrane induced by IGF-1; however, the SC-79 independent cytosolic activation of Akt due to blockage effect by ethanol was not reversed. (**F**) Ly294002 treatment did not suppress the survival and proliferation of BMSCs. Values are shown as mean ± SD (*n* = 3).

The membrane interaction and phosphorylation of Ser473 and Thr308 are required for Akt activation. Cytosolic Akt is recruited to the plasma membrane by the interaction between the PH domain and PIP3 generated by PI3K upon growth factor stimulation [17–19]. To further explore the interaction between ethanol and phosphorylation sites Ser473 and Thr308, we added a PI3K inhibitor, Ly294002, to the medium to mimic the effects of ethanol. Upon ethanol treatment, only the phosphorylation level of Ser473 was impaired, and SC-79 treatment was sufficient to rescue both impaired Ser473 phosphorylation and downstream kinase GSK3β phosphorylation (Figure 3A). The status of p-Akt-Thr308 was not decreased by ethanol treatment (Figure 3A). However, Ly294002 treatment inhibited IGF-1 stimulated Akt-Ser473 and Akt-Thr308 phosphorylation, and phosphorylation at Akt-Ser473 was partly rescued by SC-79. As a kinase downstream of Akt, GSK3β was largely reactivated (Figure 3A). Meanwhile, β-catenin and COL1 levels as well as calcium nodules in hBMSCs were also rescued by SC-79 treatment (Figure 3B, 3C, 3D). The translocation of Akt from the cytoplasm to the plasma membrane is a prerequisite for Akt activation [19]. Hence, we used confocal microscopy to image the localization of Akt in BMSCs. The translocation of Akt from the cytoplasm to the plasma membrane, an event induced by IGF-1, was largely blocked by ethanol or Ly294002 treatment compared with IGF-1 alone treatment. However, when the cells were treated with the combination of SC-79, the blockage effect of ethanol was not reversed (Figure 3E), indicating that the cytosolic activation of Akt is dependent on SC-79. In addition, treatment withLy294002 did not decrease the proliferation of hBMSCs, as showed in Figure 3F.

### GSK3β inhibitors rescues suppressive effect of ethanol via Akt/GSK3β/β-catenin axis

**Figure 4:**
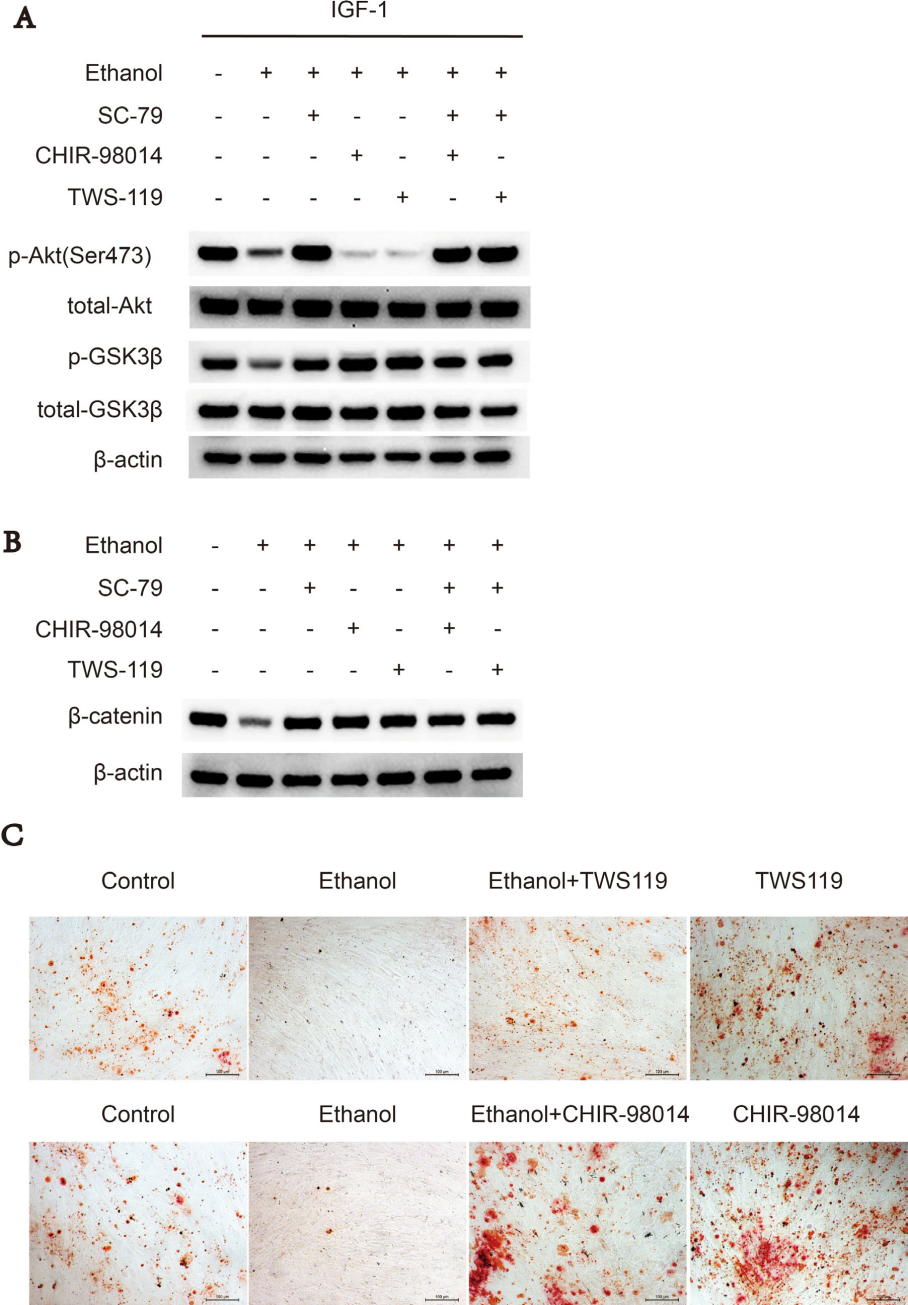
GSK3β inhibitors rescues suppressive effect of ethanol via Akt/GSK3β/β-catenin axis (**A**) CHIR-98014 and TWS119 rescued the ethanol-induced suppressive effect on p-GSK3β and β-catenin in BMSCs. The western blot shows p-Akt-Ser473, total-Akt, p-GSK3β, total-GSK3β. (**B**) Western blot of β-catenin after 72 hours. β-actin served as internal reference. (**C**) CHIR-98014 and TWS119 rescued the anti-osteogenic effect of ethanol in BMSCs, as shown by Alizarin red staining.

Since ethanol-induced Akt deactivation results in the activation of downstream kinase GSK3β and subsequent decreased β-catenin, we used two inhibitors of GSK3β, CHIR-98014 and TWS119, to abolish this inhibitory effect of ethanol. Western blot experiments showed that either CHIR-98014 or TWS119 (Figure 4A, 4B) treatment reversed the ethanol-induced decrease in p-GSK3 and β-catenin levels, while not activating Akt kinase activity. Alizarin red staining showed similar results; namely, that treatment with CHIR-98014 or TWS119 could abolish the anti-osteogenic effects of ethanol on BMSCs (Figure 4C).

### Reactivation of Akt by SC-79 prevents the development of alcohol-induced ONFH in rats

**Figure 5:**
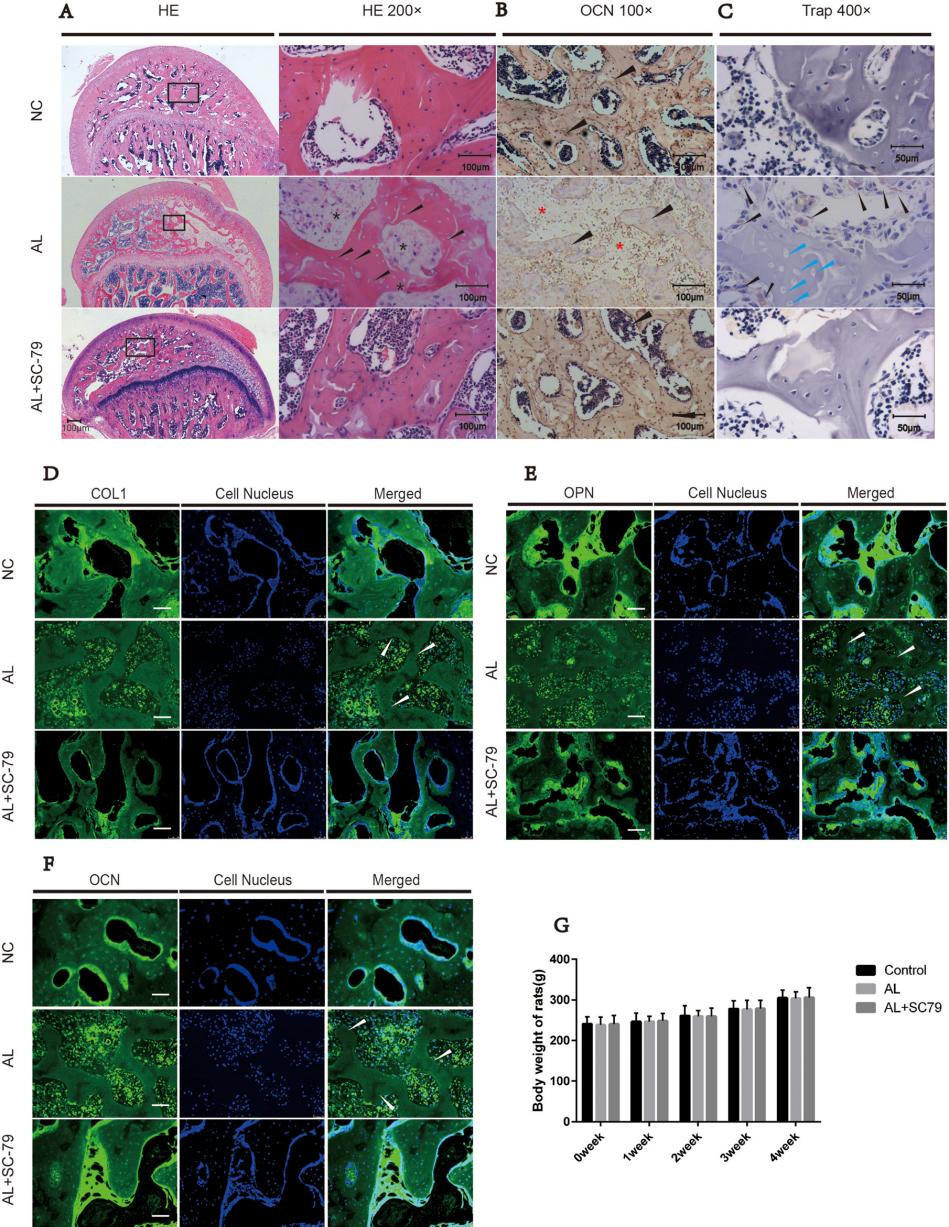
Histological findings (**A**) H&E staining of the femoral head revealed obvious osteonecrosis in the AL group. Empty lacunae in subchondral trabeculae (black arrow) with surrounding necrosis of bone marrow cells (black star) were present in the AL group while only few empty lacunae were detected in the AL+SC-79 group. (**B**) Immunohistochemical staining for OCN. Fewer trabeculae (black triangle) were positive for OCN in the AL groups while more OCN-positive trabeculae were observed in the AL+SC-79 group. (**C**) TRAP staining for osteoclast cells in femoral heads. Osteoclast cells (black triangles) were increased in the AL group. Empty lacunae (blue triangles) were also detected in the trabeculae of the AL group. (**D**–**F**) Immunofluorescent staining for OCN, OPN and COL1 in each group revealed fewer OCN, OPN and COL1 positive subchondral trabeculae in the AL group while more positive trabeculae were observed in the AL+SC-79 group. The white triangles indicated the empty lacunae. (**G**) The average weight of rats in each group was measured and expressed as mean ± SD. (*N* = 20 for each group).

We established an alcohol-induced ONFH model in rats by feeding them Lieber-Decarli ethanol-containing liquid [27, 28]. Osteonecrotic changes of the femoral head were assessed by histopathological examination after the rats received an alcohol-containing liquid diet and/or SC-79 intraperitoneal injections. ONFH was identified as diffuse empty lacunae or pyknotic nuclei present in bone trabeculae, accompanied by surrounding bone marrow cell necrosis, as previously reported [28–30]. After six weeks of alcohol administration, the subchondral area in the femoral heads was occupied by empty lacunae with surrounding bone marrow cell necrosis (Figure 5A). The number of bone trabeculae was remarkably decreased and osteonecrotic lesions were multi-aggregated. On the other hand, fewer pathological changes were observed in the AL+SC-79 group and no apparent histopathological changes were found in the NC group (Figure 5A).

Histological experiments including immuno fluorescence staining and histochemical staining were also carried out. OCN, OPN and COL1 are osteogenesis-related markers expressed during osteogenic differentiation and mineralization [31]. We measured the expression of such markers in the subchondral area of the femoral head by immunofluorescence staining as well as by histochemical staining (Figure 5B, Figure 5D–5F). Our results showed the positive staining of COL1, OCN and OPN was significantly decreased in AL group while increased in AL+SC-79 group. This indicated decreased osteogenic activity by alcohol treatment and increased osteogenesis by SC-79 co-administration (Figure 5D–5F). In addition, we also observed typical ONFH lesion including diffuse empty lacunae and significantly fewer cell nuclei in the trabecular bone in the AL group (white triangle) than in that in NC/ AL+SC-79 group. The findings were consistent with our H&E results.

Based on histological findings, 14 out of 20 rats in the AL group were classified as ONFH, while only four out of 20 rats presented mild osteonecrosis in the AL+SC-79 group (Table 1). No rat in the control group had ONFH. In addition, TRAP staining was performed to assess osteoclast activity in the femoral head (Figure 5C). The results showed a substantially greater number of osteoclast cells present in alcohol-treated rats compared to the NC and AL+SC-79 groups, especially at the boundary between necrosis and the remaining trabeculae in the subchondral bone. No osteoclast cells were observed in the region of massive osteonecrosis.

**Table 1:** The incidence of alcohol-induced ONFH

	NC	AL	AL+SC79
ONFH incidence	None	16/20^*, #^	5/20

The incidence of osteonecrosis of femoral heads in each group (*N* = 20 for each group.*, significant difference AL vs. NC group, *p* < 0.05; #, significant difference AL vs. AL+SC-79 group, *p* < 0.05).

**Figure 6:**
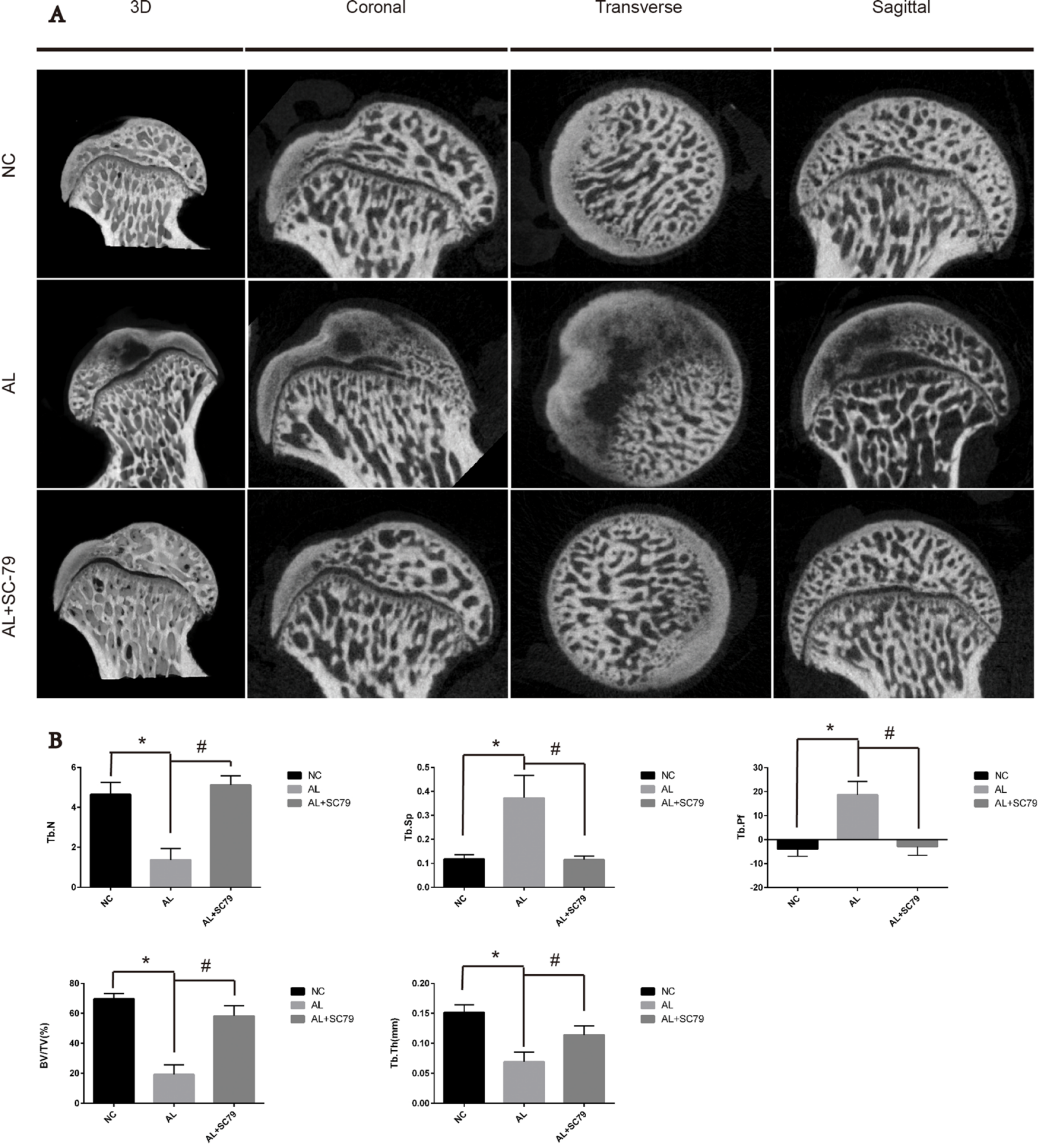
Micro-CT scanning and analyses (**A**) Micro-CT scanning images of the femoral head divided by group and section. Significant less subchondral trabecular bone was observed in the AL group compared with the NC group while more was observed in the AL+SC-79 group. (**B**) Values are represented as mean ± SD. (*N* = 5 for each group; *significant difference between the AL and the NC groups, *p* < 0.05; ^#^significant difference in the AL+SC-79 compared to the AL group, *p* < 0.05). BV/TV: bone volume/tissue volume; Tb.Th: trabecular thickness; Tb.Sp: trabecular separation; Tb.N: trabecular number; Tb.Pf: trabecular pattern factor.

Next, we used micro-CT scanning to quantitatively and qualitatively evaluate bone tissues within the femoral head after six weeks of treatment. 16 out of 20 rats in the AL group showed visible signs of ONFH, while only five out of 20 rats in the AL+SC-79 group had mild ONFH (Figure 6A), which was consistent with our histological data. No rat showed osteonecrosis in the NC group. Images of micro-CT scanning supported our results from previous experiments on alcohol-induced ONFH in rats. Quantitative analyses of micro-CT parameters further confirmed the effect of alcohol and the efficacy of SC-79 in preventing alcohol-induced ONFH in rats. As shown in Figure 6B, several parameters including BV/TV ratio, Tb.Th and Tb.N in the AL group were reduced compared to those in the NC group (*p < 0.05). Supplementation with SC-79 significantly increased the BV/TV ratio, Tb.Th and Tb.N of the femoral head compared to those in the AL group (^#^*p* < 0.05).

### Trends of body weight in rats

The average weights of rats in the NC, AL and AL+SC−79 groups were 240.8 ± 18.0 g, 238.0 ± 19.1 g and 240.5 ± 21.4 g, respectively, at the start of the experiment; and 305.3 ± 18.6 g, 303.5 ± 16.63 g, 305.8 ± 24.3 g upon sacrifice (Figure 5G). No significant difference in body weight was observed between the three groups and all rats were active and healthy.

## DISCUSSION

Alcohol is one of the leading factors of ONFH. However, in human alcoholics and in experimental animal studies, the molecular mechanisms underlying alcohol-induced ONFH are still not clear. The Lieber-DeCarli diet was first developed by Lieber *et al*. in 1982 to produce an animal model of alcohol-related diseases [32–35]. A recent study by Okazaki *et al*. showed that ONFH developed in male Wistar rats fed with a 5% ethanol-containing Lieber-DeCarli diet for 6 weeks [28]. Thus, we designed our *in vivo* experiment based on this animal model to mimic human alcohol consumption patterns. ONFH changes in such rat model were identified as diffuse empty lacunae or pyknotic nuclei present in bone trabeculae, accompanied by surrounding bone marrow cell necrosis, as previously reported [28–30].

It has been reported that steroid-induced ONFH is often accompanied by a significant reduction of osteogenesis [36–38]. In this study, we found that osteogenesis was also dramatically inhibited in the femoral heads of alcohol-induced ONFH rats, as indicated by the results of OPN, OCN and COL1 immunofluorescence staining and micro-CT analyses. Moreover, we demonstrated that ethanol treatment could markedly reduce the osteogenic differentiation and expression of osteogenesis-related genes in BMSCs *in vitro*, which further confirmed the suppressive effect of ethanol on osteogenesis.

A recent study by Chen *et al*. [11] demonstrated that ethanol could decrease GSK3β phosphorylation and protein levels of β-catenin, and impair osteogenic differentiation in BMSCs. Akt is a kinase upstream of GSK3β and β-catenin that can promote osteogenesis [14, 23, 24, 26, 39]. Therefore, we hypothesized that ethanol targets Akt, blocking osteogenic differentiation in BMSCs and resulting in alcohol-induced ONFH.

In this study, we found that interaction of Akt with the plasma membrane and the phosphorylation of Akt at Ser473 were disrupted by ethanol in BMSCs, contributing to the inhibition of osteogenesis. Interestingly, this effect could be reversed by SC-79 both *in vivo* and *in vitro*. Our observed inhibition of Akt activation by ethanol agrees with previous results by Huang *et al*. [40]. We also found that after ethanol treatment, GSK3β phosphorylation and β-catenin protein levels were reduced, indicating that the Akt/GSK3β/β-catenin signaling pathway was inhibited by ethanol. Similar results were previously reported by Chen *et al*. [11].

**Figure 7:**
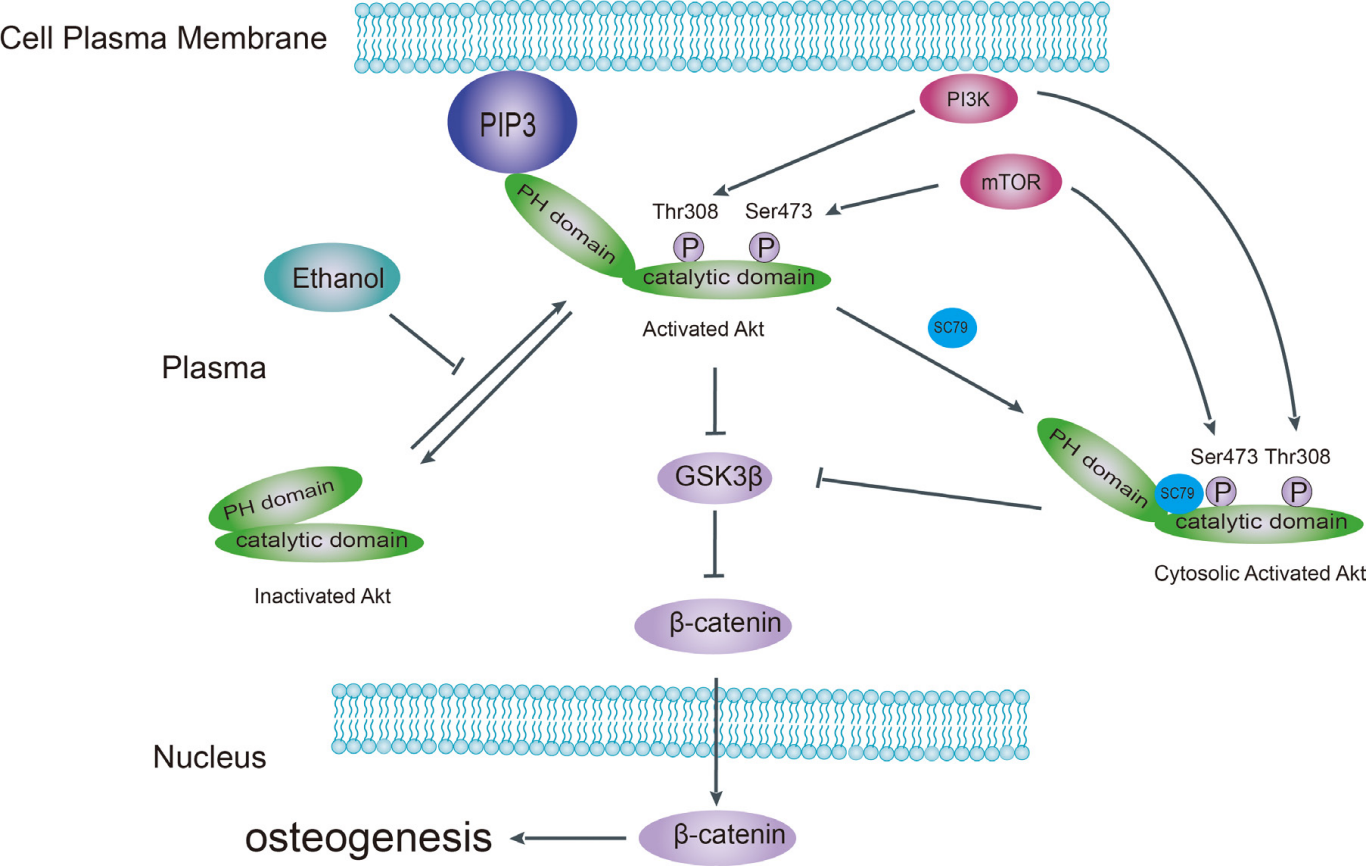
Schematic model of the pathological mechanism of alcohol-induced ONFH Ethanol impairs Akt signaling by disrupting the interaction of Akt with the plasma membrane, disturbing the interdomain conformational change of Akt, which normally exposes Thr308 and Ser473 for subsequent phosphorylation and activation. Deactivation of Akt results in activation of downstream kinase GSK3β and subsequent increased β-catenin degradation. SC-79 blocks the interaction of Akt with the plasma membrane while inducing a conformational change in cytoplasmic Akt in the Akt/GSK3β/β-catenin signaling pathway. PIP3, phosphatidylinositol 3,4,5-trisphosphate; PH, pleckstrin homology; mTOR, mammalian target of rapamycin; GSK-3β, glycogen synthesis kinase-3β.

Akt is a kinase in the PI3K pathway that promotes bone tissue development [14]. Upon upstream stimulation by activators such as IGF-1, Akt is recruited to the plasma membrane (Figure 7) via the interaction between its PH domain and PIP3, resulting in a conformational change that exposes Thr308 and Ser473 to subsequent phosphorylation. In our study, we found that the phosphorylation pattern of Akt at Thr308 and Akt-Ser473 was altered in ethanol-treated BMSCs. Ethanol reduced the abundance of p-Akt-Ser473, which might explain the observed decrease in osteogenic activity both *in vivo* and *in vitro*. We observed that the Akt activator SC-79 could reverse such effect. Furthermore, ethanol treatment inhibited the recruitment of Akt to the plasma membrane in BMSCs. However, SC-79 could not reverse the inhibition of Akt translocation, suggesting that SC-79 reactivated Akt molecules in the cytoplasm, independently from Akt recruitment to the plasma membrane [41]. As downstream targets of Akt, ethanol also inhibited GSK3β and β-catenin by suppressing Akt phosphorylation. We used several GSK3β inhibitors to rescue GSK3β and downstream β-catenin after ethanol treatment (Figure 4A–4C).

In our results, p-Akt-Thr308 was not decreased by ethanol treatment in BMSCs. This was surprising, since one would expect that both p-Akt-Thr308 and p-Akt-Ser473 would be similarly decreased upon Akt inhibition by ethanol. This effect might be directly attributable to ethanol or its metabolic products, such as those occurring during glucose metabolism [42, 43] or endoplasmic reticulum stress (ER stress) [44–47], which could result in site-specific activation of Akt. Contrary to our observations, a previous study by Liu *et al*. [48] suggested that chronic ethanol exposure increased p-Akt in mice BMMCs *in vitro*. The discrepancy might be attributable to the difference in the species of cells used, phosphorylation sites of Akt kinase detected, or the duration of ethanol treatment. We noted that ethanol has an inhibitory effect on Akt/GSK3β/β-catenin signaling while enhanced cell proliferation. This might because ethanol activates signaling pathways associated with cell proliferations, including mTOR signaling [49] and MAPK signaling [50].

In our *in vivo* experiments, SC79 was co-administrated with ethanol in SD rats. We observed lower ONFH incidence and higher OCN, OPN and COL1 expression in subchondral bone in the AL+SC79 group compared to controls. In addition, SC79 appeared to be a relatively safe drug. No changes in body weight, survival rate, appearance, and behavior in rats were observed after SC79 treatment (0.2mg/ kg/d).

Taken together, as summarized in Figure 7, we believe that ethanol-triggered anti-osteogenic effects stem from the dephosphorylation of Akt-Ser473 and the inhibition of the Akt/GSK3β/β-catenin signaling pathway. These effects of ethanol contribute to alcohol-induced ONFH. Finally, SC-79 treatment to reactivate Akt could be tested as therapeutic method to prevent alcohol-induced ONFH.

## MATERIALS AND METHODS

This study was approved by the Institutional Ethics Review Committee at Shanghai Sixth People's Hospital. All experiments on BMSCs were carried out in accordance with approved guidelines and regulations.

### Cell culture

Human bone mesenchymal stem cells (hBMSCs) were obtained from the femoral head of donors who underwent hip arthroplasty surgery as described previously [51]. The BMSCs were cultured with α-MEM (Gibco BRL, Grand Island, NY) supplemented with 10% FBS (Invitrogen, Carlsbad, CA), 1% of each penicillin and streptomycin (Gibco, Carlsbad, CA) and incubated at 37°C in 5% CO_2_. Passage of BMSCs was performed when they reached 70–80% confluence. The BMSCs obtained after four to six passages were used in all experiments. BMSCs were then treated with ethanol at the concentrations of 10 mM, 25 mM, 50 mM or 100 mM and with other reagents for the periods of time indicated in *Results*. The culture medium was changed every 24 hours and the culture plate was sealed with transparent tape and parafilm to reduce the evaporation of ethanol from the culture medium. The culture medium was saturated with oxygen and CO_2_ in the incubator for 4 h before changing the culture medium after the long-term culture with ethanol [11, 25].

### Osteogenic induction

BMSC differentiation was induced 48 h after the cells were seeded on culture plates. For osteogenic differentiation, the basal culture medium was supplemented with 10^–2^ M β-sodium glycerophosphate, 50 μg/mL L-ascorbic acid and 10^–7^ M dexamethasone. The osteoblast differentiation medium (osteogenic medium) was changed every 24 hours.

### The alizarin red staining

To measure osteogenic ability, 1 × 10^4^ cells per well were seeded in 48-well plate and grown to 90% confluence within 48 h. Then the culture medium was replaced OB medium was renewed every day. After osteogenic induction for 14 days, cells in 48-well plates were fixed with 4% paraformaldehyde for 15 min then washed with PBS and stained with 40 mM alizarin red working solution for 10 min. After being rinsed twice with PBS again, images of cells were captured using a light microscope.

### Cell toxicity and proliferation

The effect of ethanol and Ly294002 on the proliferation of BMSCs was assessed using a cell viability assay (Cell Counting Kit-8 (CCK-8), Dojindo Molecular Technologies, Inc., Japan). Briefly, BMSCs were seeded in 96-well plates at an initial density of 5 × 10^3^ cells/well and cultured in different concentrations of the ethanol (10 mM, 25 mM, 50 mM or 100 mM) or Ly294002 (50 μM) for 1, 3, 5 and 7 days. Then 20 ml of CCK-8 solution and 180 ml of culture medium were added to each well at each time point and incubated for 2 h at 37°C. Then, aliquots (100 ml) were taken from each well and transferred to another 96-well plate. The absorbance of the samples was measured at 450 nm with a spectrophotometric microplate reader (Bio-Rad 680, USA). The results were expressed as the optical density of the aliquots minus the absorbance of the blank wells.

### Confocal microscopy

BMSCs were seeded on 0.1% gelatin-coated glass coverslips placed in a 6-well plate and allowed to adhere for 48 h. We incubated cells, which had been serum-starved (0.1% serum), for 3 h with IGF-1 (100 ng/ml), ethanol (50 mM), SC-79 (10 μM) and/or Ly294002 (50 μM) for the indicated times. Cells were fixed with 4% (wt/vol) paraformaldehyde for 20 min, permeabilized with 0.3% Triton X-100 in PBS for 15 min, and incubated with primary antibodies against Akt at 4°C overnight. After being washed with PBS three times, the cells were incubated for 1 h with Alexa fluor^™^ 488 secondary antibody (Invitrogen) and DAPI for nuclear staining. Then the cells were rinsed with PBS and immunofluorescence images were captured using a Nikon confocal microscope and analyzed using Nis-Elements Viewer software.

### RNA isolation and gene expression analysis

The mRNA was extracted from BMSCs using Trizol-up (TransGen Biotech). The RT reaction was performed using Easyscript one-step gDNA Removal and cDNA Synthesis Supermix (TransGen Biotech). The qRT-PCR was carried out using TransStart Tip Green qPCR SuperMix (TransGen Biotech), forward and reverse primers (BioTNT) and cDNA. Subsequent real-time PCR analysis was carried out using an ABI 7900 HT Sequence Detection System. The relative amounts of gene expression were quantitated using a standard curve and normalized to β-actin mRNA expression. The forward and reverse primers of cDNAs were designed as follows: OCN,forward:5′-GAC CAC ATC GGC TTT CAG GA-3′ and reverse 5′-CCA GCA GAG CGA CAC CCT A-3′, RUNX2, forward:5′-TAA TCT CCG CAG GTC ACT AC-3′and reverse:5′-CTG AAG AGG CTG TTT GAT G-3′, β-actin, forward:5′ AAG GTG ACA GCA GTC GGT T-3′ and reverse:5′-TGT GTG GAC TTG GGA GAG G-3′.

### Western blotting

The proteins were extracted from BMSCs using a cell lysis buffer supplemented with proteinase inhibitor and then centrifugation at 14000 g for 15 min. The amount of protein was measure by BCA protein assay kit (Cell Signaling Technology, Danvers, MA). 20 μg of protein were resolved on SDS-PAGE gels then transferred to PVDF membranes. The protein was blocked with 5% milk in Tris-buffered saline 0.1% Tween (TBST). Then the membrane was incubated with primary human polyclonal antibody. Primary antibodies (β-catenin, GSK3β, p-GSK3β, pan-Akt and p-Akt-Ser473, p-Akt-Thr308) were provided by Cell Signaling Technology. Then the membrane was incubated with an anti-rabbit and anti-mouse secondary antibody (Cell Signaling Technology). After chemiluminescence, LEICA DM 4000 was used to detect the target bands. The protein levels were normalized by β-actin (Cell Signaling Technology).

### Inhibitors and agonists

SC-79, Ly294004, TWS199 and CHIR-98014 were purchased from Selleck (Shanghai, China). hBMSCs were cultured in medium containing specific concentrations of SC-79 (10 μM), Ly294004 (50 μM), TWS199 (10 μM) and CHIR-98014 (10 μM). Relative gene expression and western blotting were then performed.

### Animal grouping and treatment

All procedures were approved by the Animal Research Committee at Shanghai Sixth People's Hospital and carried out in accordance with the approved guidelines. Sixty 8-week-old healthy male Sprague-Dawley rats were used in this study. The rats were randomly divided into three equally sized groups: [1] control group (normal group, NC); [2] alcohol group (AL), and [3] alcohol and SC-79 group (AL+SC79). Animals in the AL and AL+SC-79 groups were fed for 6 weeks with a modified Lieber-DeCarli liquid diet (FBSH, Shanghai, China), which contains 5% (weight/volume) ethanol (36% of daily calories), fat (38% of daily calories) and protein (17% of daily calories). The control rats were fed with the Lieber-DeCarli diet without ethanol (the ethanol was substituted with maltodextrin, which provides the same amount of calories) in the same manner as the AL groups. All rats were fed the Lieber-DeCarli liquid for one week prior to the start of the experiment to adapt to the liquid diet. To minimize the difference in results due to food intake, each rat was weighed and the volume of the liquid consumed per rat was measured every day. To ensure all group of rats consumed the same amount of daily calories, the intake of liquid diet was limited to the lowest amount consumed by any group of the rats one day before. All sixty rats had access to liquid diet *ad libitum* and maintained good health throughout the study. The meals were prepared freshly on a daily basis. All rats in all groups were sacrificed under anesthesia at the sixth week and left femurs were immediately excised and fixed for 24 h in PBS containing 4% paraformaldehyde.

### Histological analysis

Proximal parts of the femur were decalcified with EDTA for one month and embedded in paraffin. Specimens of the femoral head were cut into 4-mm-thick sections. Femoral head specimens were stained with hematoxylin & eosin (H&E) for the evaluation of subchondral trabecular structure. The femoral heads were then deparaffinized, antigen retrieved and incubated with anti-OPN, anti-OCN and anti-COL1 (Abcam, Cambridge, MA) primary antibody, then treated with secondary antibodies. Osteoclast activity was performed on decalcified femoral heads embedded in paraffin. Serial sections from paraffin blocks (~7 mm thick) were stained for tartrate-resistant acid phosphatase (TRAP) activity and counterstained with alcian blue.

### Micro-CT scanning

Micro-CT scanning of the femoral heads of rats was performed with a micro-CT scanner (Skyscan 1176; Kontich, Belgium) set at9-micron voxel size. Image acquisition was performed at 35 kV of energy and 220 mA of intensity. The images were processed with CTVol software and reconstructed. Then the trabecular bone volume fraction (BV/TV), trabecular thickness (Tb.Th), trabecular number (Tb.N), trabecular pattern factor (Tb.Pf) and trabecular separation (Tb.Sp), were measured from the reconstructed images.

### Statistical analysis

GraphPad Prime 6 was used to analyze the data in all groups. The data were reported as mean ± standard deviation (SD). The comparisons between groups were carried out with Student's *t* test. Statistical differences were considered as significant at *p* < 0.05.
